# Oral Rehabilitation of Oral Cancer Patients Using Zygomatic Implant-Supported Maxillary Prostheses with Magnetic Attachment: Three Case Reports

**DOI:** 10.1155/2018/1694063

**Published:** 2018-09-16

**Authors:** Hisashi Ozaki, Hiromasa Sakurai, Yukie Yoshida, Hideyuki Yamanouchi, Mitsuyoshi Iino

**Affiliations:** ^1^Department of Dentistry, Oral and Maxillofacial Surgery, Yamagata Prefectural Central Hospital, Yamagata, Japan; ^2^Department of Dentistry, Oral and Maxillofacial-Plastic and Reconstructive Surgery, Faculty of Medicine, Yamagata University, Yamagata, Japan; ^3^Department of Dentistry, Oral and Maxillofacial Surgery, Nihonkai General Hospital, Yamagata Prefectural and Sakata Municipal Hospital Organization, Sakata, Japan

## Abstract

Maxillectomy for malignant tumor often results in a maxillary defect and serious oral dysfunction. A prosthesis is usually provided for postoperative oral rehabilitation of such patients with maxillary defects. However, the further the resected region extends, the less stable the prosthesis becomes, due to insufficient bone and tooth support. Therefore, in many cases, conventional resection dentures may not be adequate to restore the oral function. Effective utilization of dental and zygomatic implants may help to restore oral function in patients with severe maxillary defects. This clinical report describes the management of three patients with severe maxillary defects following cancer ablative surgery who were rehabilitated using maxillary prostheses with magnetic attachments supported by dental and zygomatic implants. Occlusal reconstruction was performed with removable prostheses supported with two or four implants and magnetic attachment. The oral function was evaluated before and after prosthodontic treatment with implants using the Oral Health Impact Profile (OHIP-14) and functional chewing score. Results indicated improvement in all cases. These findings show that quality of life (QOL) and oral function were improved.

## 1. Introduction

Maxillectomy is performed for radical treatment of maxillary malignant tumors leading to serious problems in mastication, swallowing, speech, and facial esthetics. Reconstruction is of paramount importance for these individuals but is often a major challenge. There are several reconstructive techniques that involve the use of vascularized or nonvascularized autogenous material or prosthetic devices with dental and/or zygomatic implants. Conventional dental implants have been used to improve the stability and retention of maxillary prosthetic obturators and to restore oral function [1, 2]. However, dental implant placement is often difficult following resection of maxillary malignant tumor because of inadequate amount of bone tissue for anchorage of the implants. As an alternative procedure, the use of zygomatic implants is effective for prosthetic rehabilitation [3–5]. The effective utilization of dental and zygomatic implants may help to restore oral function in patients with severe maxillary defects.

Here, we describe the management of three patients who underwent extensive maxillary resection resulting in huge maxillary defects, followed by the introduction of maxillary prostheses with magnetic attachment using dental and zygomatic implants.

## 2. Case Presentation

Between October 2012 and November 2013, three patients with maxillary defects following resection of malignant tumor were recruited. The clinical findings of these patients are presented in Table 1. Type of maxillary defect was defined based on the classification by Brown et al. [6]. The implant systems used were Brånemark System® MK-III and Zygoma TiUnite (Nobel Biocare, Zurich, Switzerland). The mean healing period until second surgery was 6.7 months (range: 6–8 months). Conventional resection dentures were initially fabricated, followed by implant-supported overdentures with magnetic attachments.

### 2.1. Case 1

**Figure 1:**
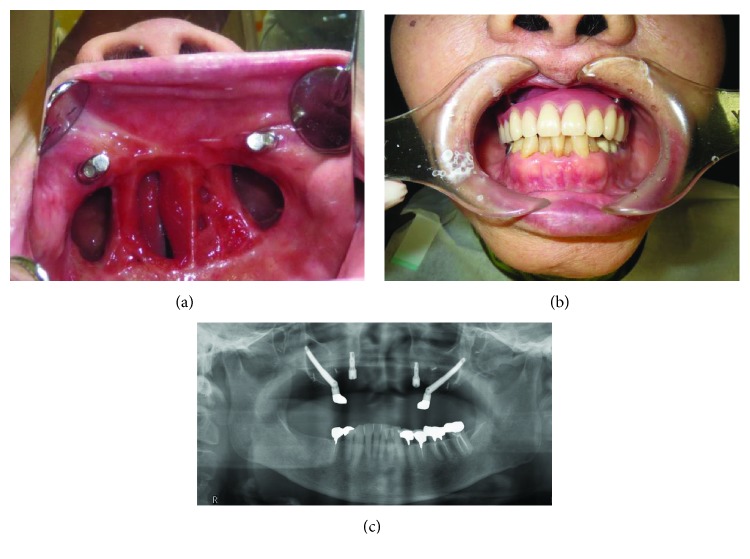
(a) Postoperative intraoral photograph (mirror image). (b) Intraoral view with the prosthesis in place. (c) Postoperative radiograph.

A 76-year-old woman with malignant melanoma of the upper gingiva underwent subtotal maxillectomy and neck dissection of the right side. Six months after tumor resection, two zygomatic implants were inserted into bilateral zygomatic bones. After another 6 months, second-stage surgery was performed and two dental implants were placed in the anterior region of the maxilla. However, the position and depth of the dental implants were inappropriate for the final prosthesis. Therefore, the two anterior implants could not be used for support. The zygomatic implants and prosthesis have remained stable for 3 years since functional loading (Figures 1(a)–1(c)).

### 2.2. Case 2

**Figure 2:**
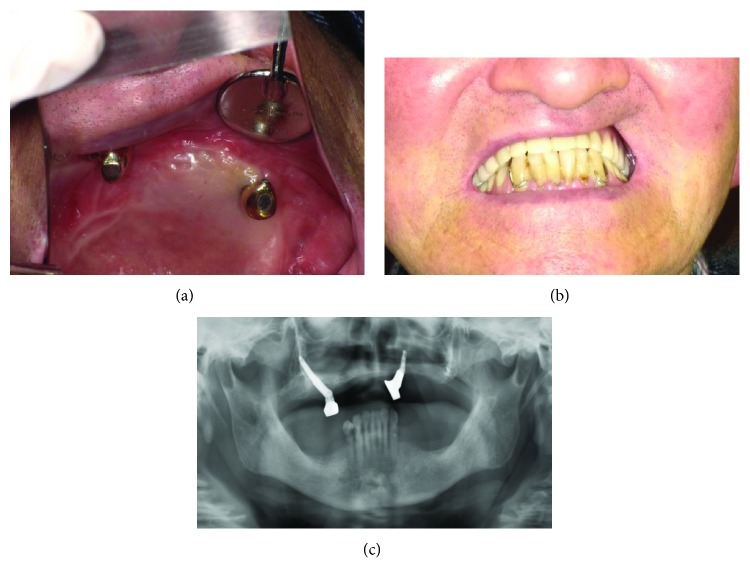
(a) Postoperative intraoral photograph (mirror image). (b) Intraoral view with the prosthesis in place. (c) Postoperative radiograph.

An 81-year-old man was diagnosed with squamous cell carcinoma of the left maxillary gingiva and underwent partial maxillectomy. Two years after tumor resection, two dental implants in the anterior maxillary region and one zygomatic implant into the right side zygomatic bone were placed. After another 6 months, second-stage surgery was performed; however, one dental implant in the anterior region had to be explanted due to loss of osseointegration. Subsequently, the implants and prosthesis have remained stable for 1 year and 6 months since functional loading (Figures 2(a)–2(c)).

### 2.3. Case 3

**Figure 3:**
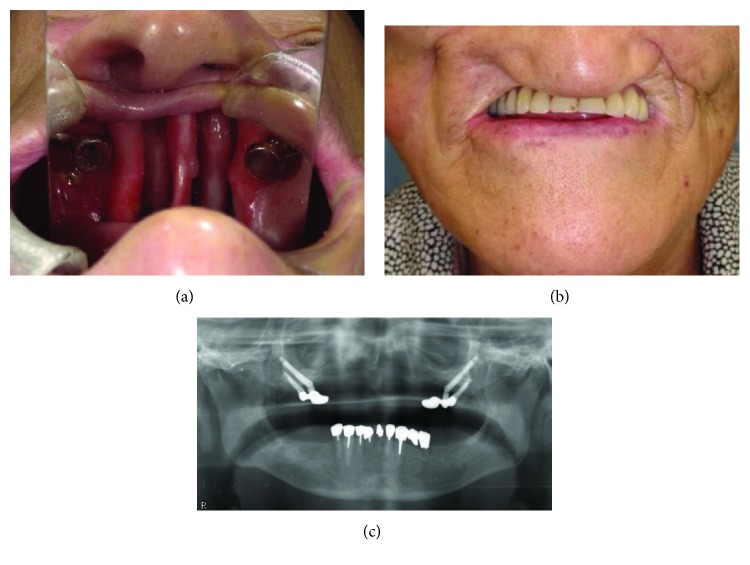
(a) Postoperative intraoral photograph (mirror image). (b) Intraoral view with the prosthesis in place. (c) Postoperative radiograph.

An 83-year-old woman had a chief complaint of difficulty in eating due to severe instability of her upper removable denture. Fifteen years ago, she had been diagnosed with malignant melanoma of the maxillary gingiva. After preoperative superselective arterial injection chemotherapy, bilateral partial maxillectomy and postoperative concurrent chemoradiotherapy were performed. Thirteen years after tumor resection, two dental implants and two zygomatic implants were placed on each side of the zygomatic bones. Two years after functional loading, the left abutment with magnetic attachments was fractured. A new abutment with magnetic attachments was fabricated, and the prosthesis is currently being used without any complications (Figures 3(a)–3(c)).

### 2.4. Evaluation of OHRQoL and Masticatory Function

Oral health-related quality of life (OHRQoL) was measured using the Oral Health Impact Profile [7] (OHIP-14) before and after prosthodontic treatment with implants. Higher scores in OHIP-14 indicate worse result. Masticatory function was assessed using an evaluation sheet for chewing function [8]. In all cases, the numerical value decreased in OHRQoL and the chewing function scores increased after prosthodontic treatment with implants (Table 2).

## 3. Discussion

Maxillary defects caused by cancer ablative surgery are commonly reconstructed with prostheses. Good functional results are reportedly attained with obturator prostheses [9–12]. However, the further the resected region extends, the less stable the prosthesis becomes because of insufficient bone and tooth support for the denture. The use of dental implants is effective in such cases. In the present study, it is obvious that the maxillary prosthesis with magnetic attachment supported by dental and zygomatic implants was effective as shown on OHRQoL. With regard to masticatory function, in Cases 1 and 3, the chewing function scores with the conventional resection denture were 20 and 45, respectively. In contrast, the scores with the maxillary prosthesis supported by the implants were 50 and 65, respectively.

Sato et al. reported that the mean score of complete denture wearers with “satisfied” was 58.7, “partly satisfied” 48.5, and “not satisfied” 32.4. These scores offer a ready explanation that the chewing function score corresponds closely to chewing satisfaction [8]. Therefore, in Case 1 and Case 3, it is thought that the chewing function is not inferior to the function of complete denture wearers. This treatment could provide the recovery of chewing function in consideration of poor environment in the oral cavity. However, in Case 2, the chewing function score showed only a slight increase, probably because there were only few remaining teeth in the mandible and the mandibular partial denture did not fit well. The limitation of the present treatment is incomplete closure of the resulting maxillary defect, such as Cases 1 and 3. For this problem, Butterworth et al. suggested a new surgical technique with the zygomatic implant perforated flap. The technique involves the use of a zygomatic implant perforated microvascular soft tissue flap (ZIP flap) for the primary management of maxillary malignancy with surgical closure of the resultant maxillary defect and the installation of osseointegrated support for a zygomatic implant-supported maxillary fixed dental prosthesis [13]. In the report, this treatment demonstrated good result for the case of the maxillary malignant. However, the treatment with free tissue transfer is very invasive. Moreover, the application of the treatment is a low-level Brown class 2b maxillectomy and limited [14]. Our cases are all very elderly people, and Cases 1 and 3 are Brown class 2d. Therefore, the ZIP flap technique is not suitable in our cases.

Various attachment systems have been successfully used with implant-supported overdentures in recent years. These systems include telescopic crowns, bars, locators, balls, and magnets. Dental practitioners and technicians generally select attachment systems based on their experience and training [15]. We selected the magnet attachment system to reduce the load to the implants. Depending on the extent of the maxillary defect, the denture tends to become larger and wider. Therefore, it was assumed that the load to the prosthesis, including implant and abutment, may increase during occlusion, compared to conventional implant-supported overdentures without the maxillary defect. Rigid retention between the denture and implant may increase the risk of prosthodontic complications, including fracture of denture, abutment, and implant. As magnetic attachments resist only vertical force and do not resist lateral force, it is thought that retention is low against lateral force compared to the other attachment. Consequently, the abutment and implant body appear to be better protected. However, in Case 3, the abutment with magnet attachment was fractured. This fracture could have occurred because the abutment was too long. Therefore, a favorable position and angle of placement of the implant are important for the prosthesis.

There is no clear consensus on the appropriate number of dental and zygomatic implants required for the implant-supported maxillary prosthesis in patients with maxillary defects. Schmidt et al. [4] presented a review of patients who underwent reconstruction using zygomatic implants after maxillectomy and found that four zygomatic implants or a combination of two dental implants and zygomatic implants were used for functional and aesthetic rehabilitation after maxillectomy. The prognosis of such treatment was acceptable. In the present study, the oral functions of 2 cases were restored by a two-implant-supported overdenture in short term. This approach will offer several advantages [3]. First, additional procedures for reconstruction of the maxilla will not be necessary in many cases. Second, the placement of implants and fabrication of the prosthesis become simple. Finally, the time required for surgery is reduced and the reduced number of implants reduces the cost [3].

These cases demonstrated that a maxillary prosthesis with magnetic attachment supported by dental and zygomatic implants is effective for patients with maxillary defects.

## Figures and Tables

**Table 1:** Detailed information on the patients.

	Case 1	Case 2	Case 3
Age/gender	76/F	81/M	83/F
Type of cancer	Malignant melanoma	Squamous cell carcinoma	Malignant melanoma
Defect	Class IIc	Class Ia	Class IIc
Location	Bilateral buccal bone	Buccal bone and anterior region	Bilateral buccal bone
Number of implants	Dental implant: 2	Dental implant: 2	Dental implant: 2
	Zygomatic implant: 2	Zygomatic implant: 1	Zygomatic implant: 2
Length and width	Dental implant: 10 mm, 3.5 mm	Dental implant: 10 mm, 3.5 mm	Dental implant: 18 mm, 4 mm
	Zygomatic implant: 40 mm, 4 mm	Zygomatic implant: 30 mm, 4 mm	Zygomatic implant: 30 mm, 4 mm
Healing period	8 months	6 months	6 months
Period of loading	3 years	1 year and 6 months	2 years
Radiation	None	None	50 Gy

**Table 2:** Evaluation on OHIP-14 and functional chewing score.

Questionnaire	OHIP-14		Functional chewing score	
	Pre	Post	Pre	Post
Case 1	45	12	20	50
Case 2	16	6	30	35
Case 3	31	18	45	65
